# Personal

**Published:** 1897-02

**Authors:** 


### Personal.

---

SIR JOSEPH LISTER, the eminent English surgeon, whose name is always associated with the application of antiseptic principles to surgical procedures, was raised to the peerage by the Queen on the first day of the new year. This is the first occasion, according to the *British Medical Journal*, on which a member of the medical profession has entered the house of lords, and we agree with our English contemporary that the profession of medicine could not wish or expect for a representative more able or distinguished. Sir Joseph's American friends will all unite in congratulations upon his elevation to the peerage and in the wish for his continued health and prosperity.

Dr. Elizabeth Blackwell, who, at the age of seventy-five, has lately published a book of autobiographical notes, entitled Pioneer Work in opening the medical profession to women, was the first woman to receive a medical diploma from any medical college in this country or Europe. Though she is a native of England, she is an American by adoption, having taken her degree and pursued the greater part of her medical practice in this country.

---

Dr. Alexander C. Abbott, professor of hygiene at the University of Pennsylvania, has been appointed chief of the bacteriological division of the bureau of health at Philadelphia, in place of Dr. B. Mead Bolton, resigned. It will be remembered that Dr. Abbott read a paper last winter before the Buffalo Academy of Medicine, that was published in the Journal for March, 1896.

---

Dr. Chauncey P. Smith, of Buffalo, has been appointed by Mayor Jewett a member of the Municipal Civil Service Commission, vice Dr. H. E. Hayd, resigned. A pressure of professional business prevents Dr. Hayd from longer holding the office in which he has served for several years. The appointment of Dr. Smith is to be commended.

---

Dr. Guy L. McCutcheon has left Buffalo to make his home in Oil City, where he will take up the practice of medicine. His many friends in Buffalo will be sorry to part with him, and all wish him every success in his new field.

---

Dr. W. E. Dignen, of Buffalo, is now in Berlin. Since leaving Buffalo, he has spent some time in London and Paris.

